# Epidemiological and clinical profiles of respiratory syncytial virus infection in hospitalized neonates in Suzhou, China

**DOI:** 10.1186/s12879-015-1155-x

**Published:** 2015-10-15

**Authors:** Lianghua Lu, Yongdong Yan, Bin Yang, Zhihui Xiao, Xing Feng, Yuqing Wang, Wei Ji, Maximillion Mize, Chuangli Hao, Zhengrong Chen

**Affiliations:** Department of Neonatology, Children’s Hospital of Soochow University, Suzhou, 215003 China; Department of Respiratory Disease, Children’s Hospital of Soochow University, Suzhou, 215003 China; Department of Clinical Lab, Children’s Hospital of Soochow University, Suzhou, 215003 China; Department of Cell Biology and Immunology, UNT Health Science Center at Fort Worth, Fort Worth, TX 76107 USA

**Keywords:** Lower respiratory tract infection, Neonate, Respiratory syncytial virus, Climate factors, Severity

## Abstract

**Background:**

This study was designed to explore the epidemiological and clinical profiles of respiratory syncytial virus (RSV) infection in neonates from the Suzhou area of China, taking into consideration how climate factors influence disease.

**Methods:**

From 2010 to 2014, nasopharyngeal aspirates (NPA) collected from hospitalized neonates with lower respiratory tract infections (LRIs) were screened for seven common respiratory viruses including RSV by direct immunofluorescence assay. Human bocavirus, human metapneumovirus, and mycoplasma pneumoniae were detected by polymerase chain reaction.

**Results:**

Of the 1803 hospitalized neonates analyzed, 20.74 % were found to be infected with RSV. Interestingly, 30 subjects were identified as being coinfected with other viruses. The rate of RSV infection was highestduring thewinter and early spring seasons; however, infection was negatively associated with monthly mean temperature (r_s_ = −0.821, *P* < 0.0001), total rainfall (r_s_ = −0.406, *P* = 0.002), and sum of sunshine (r_s_ = −0.386, *P* = 0.001). Monthly mean temperature was the only independent factor associated with RSV activity, as determined using multivariate regression analysis. Compared with non-RSV neonates, neonates with RSV infection presented more frequently with tachypnea,moist rales, and abnormal chest X-rays requiring supplemental oxygen and extended hospitalization postpartum. Neonatal admittance into the NICU was determined based on prematurity and coinfection with other viruses; two independent risk factors for RSV disease, as determined by multivariate logistic analysis.

**Conclusions:**

Important as a major cause of LRIs in hospitalized neonate, we found that the subtropical climate of the Suzhou area was associated with RSV activity. The identified risk factors ofsevere disease in neonates with RSV infection should be taken into consideration when implementing disease health interventions.

## Background

Respiratory syncytial virus (RSV) is a leading cause of acute respiratory tract infections in infants and young children, with an estimated 3.4 million hospitalizations and 0.6 million deaths reported annually worldwide [1, 2]. Because of the limited antibody response and reduced concentration of passively acquired maternal antibodies in neonates, preterm neonates are highly susceptible to RSV [3]. In addition, with the lacki of an effective vaccine, neonates and newborns are more prone to lower respiratory tract infections (LRIs) caused by RSV. Accounting for 81 % of all pneumonia cases diagnosed as viral community-acquired pneumonia (CAP), RSV has been associated with significant morbidiy and mortality among neonates [4].

Few studies have focused on the burden of neonatal RSV infection in China. However, past studies have reported on the prevalence and clinical characteristics of RSV infection in children over 2 months old. Therefore, the purpose of this study was to explore the epidemiological and clinical profiles of neonates presenting with RSV infection in Suzhou China and paying close attention to the association between infection and climate factors.

## Methods

### Study population

This study was conducted at Children’s Hospital of Soochow University, the only comprehensive tertiary children hospital serving most young children in Suzhou. From January 2010 to December 2014, nasopharyngeal aspirates (NPA) were collected within 24 h from all hospitalized neonates (≤28 days) who were diagnosed with LRIs (either bronchiolitis or pneumonia). Lower respiratory tract infection was defined as: the presence of wheezing, tachypnea, chest retractions, abnormal auscultatory findings (wheezing and crackles), the presence of fever, and radiologic evidence indicative of a LRI. Chest radiography was performed using standard equipment and radiographic techniques, and reviewed by the radiologists in digital format. Low birth weight was considered < 2500 g for the present study.

All NPAs were immediately sent to the laboratory to assess the presence of RSV as determined by direct immunofluorescent assay (DFA). This study was approved by the Ethics Committee of Children’s Hospital of Soochow University, and consent forms were obtained from the guardians for all children enrolled in this study.

### Data collection

Data on patient demography, clinical symptoms, complications, underlying diseases (i.e. congenital heart disease, bronchopulmonary dysplasia, bronchopulmonary malformation, Down’s Syndrome) and laboratory findings (including radiographic outcomes) were obtained from the hospital medical records system.

### Detection of common respiratory pathogens

Seven common respiratory viruses were detected by DFA (D3 Ultra DFA respiratory virus screening and identification kit, Athens, Ohio, USA). Nasal passage aspirates, were collected as previously described, with sample from each neonate divided into two sections for detection of viral species by DFA and PCR, respectively. All freshly made slides with smears of exfoliated cells were used for viral antigen detection (RSV, influenza virus (IV-A and IV-B), parainfluenza virus (type 1, 2, 3) and adenovirus) as described previously [5].

Human bocavirus and metapneumovirus, as well asMycoplasma pneumoniae, were detected by PCRs as previously described [6]. Briefly, the other half of a patient NPA was used for DNA or RNA extraction. Samples were centrifuged at 12,000 × g for 5 min, followed by extraction of DNA and RNA from a 400-μl sample using DNA-EZ Reagents (Sangon Biotech, Shanghai, China) or TRIzol Reagent (Life Technologies, Carlsbad, USA) in accordance with the manufacturer’s instructions. A final volume of 200-μl, containing either DNA or RNA, was eluted anddivided into two aliquots for detection of human bocavirus and Mycoplasma pneumoniae gene amplification via realtime PCR. RNA sample was used for human metapneumovirus gene amplification via reverse transcription PCR.

### Climate data collection

Climate data (monthly mean temperature (°C), relative humidity (%), total rainfall (mm), sum of sunshine (h), and wind velocity (m/s) were provided by the Meteorological Bureau of Suzhou at longitude 120°6’ east and latitude 31°3’ north, which is located 8 km away from the Children’s Hospital of Soochow University.

### Statistical analysis

Statistical analysis was performed using SPSS v.17.0 for Windows (SPSS Inc., Chicago, IL). Discreet data were analyzed using the Chi-square test. Continuous data were analyzed using the T-test for normally distributed data, while the Kruskal-Wallis test was used to analyze data that failed to show a normal distribution. The association between RSV cases and climate factors were analyzed using the Spearman correlation test. To detect interactions among the climate factors multivariate regression analysis was used. All tests were two-tailed with *P* < 0.05 considered statistically significant.

## Results

### Incidence and demography of all neonates with RSV infection

From 2010 to 2014, a total of 1803 NPAs from neonates hospitalized with LRTs were obtained to detect viruses and Mycoplasma pneumoniae. Of all 1803 caseswith LRTs, 374 cases were confirmed with RSV, 75 with IV-A, 45 with M. pneumoniae, 15 with PIV-3, 10 with IV-B, 10 with ADV, 5 with HBoV, and 3 with hMPV. Accounting for 20.74 % (374/1803) of all LRT cases, RSV was the most commonly identified respiratory virus in our samples. A total of 163 cases were identified as being infected with other viruses not listed here; this includes 30 cases in which patients were coinfected with RSV and another respiratory patohgen. As shown in Table 1, IV-A was the most common virus that established coinfection with RSV in neonates.

**Table 1:** Distribution of co-infection with RSV

Co-infection distribution	Positive number	Percent
Co-infection with RSV		
Influenza virus A	12	40.0
*Mycoplasma pneumoniae*	8	26.7
Influenza virus B	5	16.7
Parainfluenza virus type 3	2	6.7
Human metapneumovirus	1	3.3
Humna bocavirus	1	3.3
Adenovirus	1	3.3
Total co-infection	30	100

*RSV* respiratory syncytial virus

**Fig. 1:**
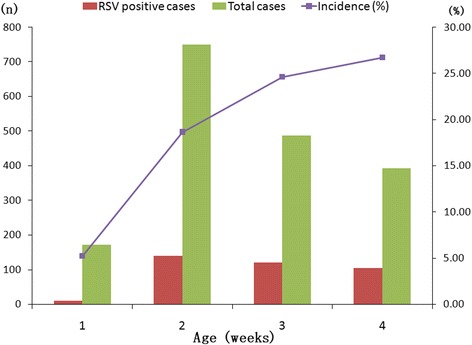
Age distribution of hospitalized neonates with lower respiratory tract infections (LRIs) related to respiratory syncytial virus

Among the 374 cases of RSV infection, the youngest neonate was 5 days old and the oldest was 28 days post-delivery; the median age was 17 days old. Age distribution of RSV-positive neonates is shown in Fig. 1. This data indicates that the incidence of RSV-positive neonates increases with age. Of all 374 cases of RSV infection, 224 (59.9 %) neonates were male yielding a male: female ratio of 1.49:1. Compared to the male: female ratio of other viral infections (1.67:1), no significant difference was found in gender between RSV-positive neonates and neonates testing positive for other viruses (*P* > 0.05). Only 28 cases (7.5 %, 28/374) were reported to have a gestational age less than 37 weeks and most of RSV-positive neonates grew to full term.

### Seasonal and monthly distribution of neonates with RSV infection

**Fig. 2:**
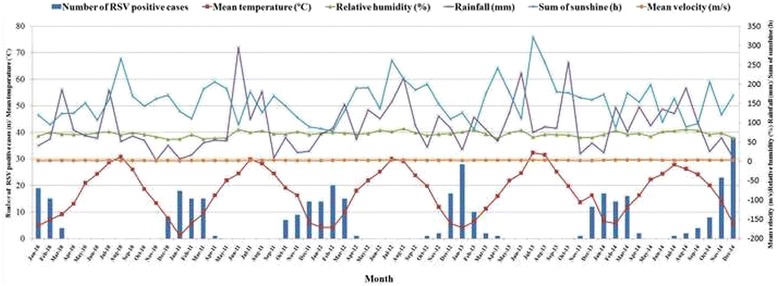
Seasonal and monthly distribution of respiratory syncytial virus (RSV) infection and associations with climate factors for a 4-year period from January 2010 to December 2014. Meteorological data was measured hourly and then average daily values were calculated. Monthly means were calculated using the daily means for temperature, relative humidity, and wind velocity. Total rain and sum of sunshine were calculated as a total measurement for the month. Spring: March to May; summer: June to August; autumn: September to November; winter: December to February

From 2010 to 2014, the annual RSV incidences were 8.2 (46/562), 18.8 (79/420), 6.8 (70/515), 13.6 (53/419), and 31.0 % (126/406) respectively. High incidence was found in the year 2014 (*P* < 0.05), especially in December. Infection with RSV mainly occurred in winter and early spring (December to May), and prevailed from November to April, with a peak from December to February each year. RSV-positive cases were rarely observed each year during summer (June to August) with theexception of the year 2014 (Fig. 2).

### Association between RSV infection and climate factors

From 2010 to 2014, the monthly mean temperature was 17.3 ± 8.8 (mean ± S.D.) °C, relative humidity was 71.0 ± 6.8 %, total rainfall was 90.1 ± 64.7 mm, sum of sunshine was 156.3 ± 50.8 h, and wind velocity was 2.2 ± 0.5 m/s. The monthly mean data for climate factors are shown in Fig. 2. Associations between RSV-positive neonates and climate factors were explored by Spearman rank correlations. Monthly mean temperature, sum of sunshine and total rainfall were significantly correlated with the number of RSV-positive neonates with LRIs (*P* < 0.05). The spearman correlation coefficients are −0.821, −0.406, and −0.386, respectively. Having the lowest seasonal temperatures of the year, RSV activity was reported to be at its highest during the winter months.

In light of intercorrelations among these climate factors, multivariateregression analysis was used to analyze the associations between the number of RSV-positive cases and our chosen climate factors. Monthly mean temperature was the only independent factor shown to be associated with the number of RSV-positive cases (Table 2).

**Table 2:** Associations between RSV incidence and climatic factors

Climate factors	Spearman rank correlation coefficients		Multivariate regression analysis	
	r_s_	*P* value	Standardized beta coefficient	*P* value
Mean temperature (°C)	−0.821	<0.0001	−0.758	<0.0001
Relative humidity (%)	−0.145	0.269	0.084	0.357
Total rainfall (mm)	−0.386	0.002	−0.096	0.311
Sum of sunshine (h)	−0.406	0.001	0.024	0.814
Wind velocity (m/s)	−0.149	0.255	0.125	0.173

*RSV* respiratory syncytial virus

### Clinical and laboratory characteristics in neonates with RSV infection

Clinical and laboratory characteristics in neonates suffering solely of RSV infection are summarized in Table 3. Compared toneonates infected with viruses, RSV-positive neonates were younger and presented more frequently with tachypnea, moist rales, and abnormal chest X-ray requiring supplemental oxygen and an extended hospital stay. Only one neonate who presented with low birthweight (1950 g) and congenital heart disease died following respiratory and heart failure. No significant difference was shown in other parameters between RSV-positive neonates and those infected with other respiratory viruses.

**Table 3:** Comparison of Clinical and laboratory characteristics between neonates with RSV and viruses other than RSV

Clinical Parameters	RSV- LRIs	Other viruses LRIs	*P* Value
	*n* = 344	*n* = 131	
Demographic data			
Age (median, IQR)	17 (12–22)	17 (13–23)	0.002
Sex (male, %)	205 (59.6)	70 (53.4)	0.224
Birth weight (mean ± std, g)	3380.5 ± 460.6	3390.8 ± 471.1	0.820
Underlying condition (*n*, %)^a^	35 (10.2)	11 (8.4)	0.558
Clinical symptoms			
Cough (*n*, %)	343 (99.7)	131 (98.5)	1.0
Fever (*n*, %)	75 (21.8)	46 (35.1)	0.003
Wheezing (*n*, %)	34 (9.9)	12 (9.2)	0.812
Tachypnea (*n*, %)	174 (50.6)	31 (23.7)	<0.001
Dyspnea (*n*, %)	68 (19.8)	17 (13.0)	0.084
Cyanosis (*n*, %)	18 (5.2)	8 (6.2)	0.708
Refusal to feed (*n*, %)	14 (4.1)	6 (4.6)	0.804
Physical examination			
Moist rales (*n*, %)	267 (77.6)	72 (55.0)	<0.001
Wheezing rales (*n*, %)	34 (9.9)	12 (9.2)	0.812
Laboratory tests			
White blood cells	9.1 ± 2.7	9.2 ± 3.0	0.777
C-reactive protein (median, IQR)	0.9 (0.3–2.6)	0.6 (0.1–2.1)	0.061
Alanine transarninase increase (*n*, %)	20 (5.8)	7 (5.3)	0.843
Creatine kinase-MB increase (*n*, %)	257 (74.7)	85 (64.9)	0.033
Abnormal chest X-ray (*n*, %)	307 (89.2)	67 (51.4)	<0.001
Theraphy			
Supplemental oxygen (*n*, %)	59 (17.2)	11 (8.4)	0.016
Mechanical ventilation (*n*, %)	15 (4.3)	3 (2.3)	0.291
Duration of hospital days (median, IQR)	10 (9–12)	8 (7–10)	<0.001

^a^Underlying conditions: Congenital heart disease; Bronchopulmonary dysplasia; Bronchopulmonary malformation; Down Syndrome. *RSV* respiratory syncytial virus, *LRI* lower respiratory tract infection, *IQR* inter-quartile ranges

In the present study, a total of 48cases were hospitalized in NICU and care in NICU was considered as a marker of severe illness. Multivariate analysis by logistic regression revealed that prematurity, coinfection with other viruses,and underlying diseases were high risk factors of NICU stay (Table 4).

**Table 4:** Risk factors of NICU stay in neonates with RSV related LRIs

Parameters	NICU	Ward	Crude OR (95 % CI)	Adjusted OR (95 % CI)	P
	*n* = 48	*n* = 326			
Sex (male, %)	28 (58.3)	196 (60.1)	0.929 (0.502–1.718)	0.692 (0.353–1.357)	0.284
Prematurity (*n*, %)	10 (20.8)	10 (3.1)	8.316 (3.252–21.265)	6.679 (2.279–19.580)	0.001
Early neonate (*n*, %)^a^	3 (6.3)	6 (1.8)	3.556 (0.859–14.719)	3.897 (0.845–17.972)	0.081
Low Birth Weight (*n*, %)	5 (10.4)	8 (2.5)	4.622 (1.446–14.771)	1.651 (0.427–6.388)	0.468
Co-infection with other viruses (*n*, %)	10 (20.8)	20 (6.1)	4.026 (1.755–9.239)	2.720 (1.047–7.065)	0.040
Underlying diseases (*n*, %)	9 (18.8)	29 (8.9)	2.363 (1.042–5.361)	2.831 (1.183–6.774)	0.019

^a^Early neonate indicates age less than one week. *NICU* neonatal intensive care unit, *OR* Odds Ratio, *RSV* respiratory syncytial virus, *LRI* lower respiratory tract infection

## Discussion

The burden of hospitalization for children with community-acquired pneumonia was highest among the very young, and RSV is the most commonly detected virus causing pneumonia [7]. Infection with RSV is associated with mortality in hospitalized infants and young children [8], especially in infants less than 3 months old [9]. Exactly which parameters contribute to the seasonality of RSV in neonates and their comparative significances are the subject of ongoing intensive debate.

Few large sample studies have focused on neonates with RSV infection. This study describes the epidemiology of RSV in neonates for four consecutive years. In Suzhou, RSV is the most commonly detected virus in hospitalized neonates with LRIs, accounting for 20.74 % of neonate respiratory infection. The incidence of RSV reported here was only slightly lower than that observed bythe study in Tunisia using DFA (23.1 %) [10]. In the present study, all neonates were < 28 days old compared to their criteria of < 35 days old in the Tunisia study. As shown in our study, the incidence of RSV-positive neonates increased with age, explaining why the incidence of RSV infection in this study was slightly lower than the Tunisia study. Interestingly, the incidence of infection in our study was higher than that seen in aneonatal medium care unit in Netherlands (1.8 %, 6/334) [11]. During the Netherlands study, information on symptomatic neonates were used regardless of whether the subjects tested positive for RSV. In constrast, only RSV-positive neonates were surveyed possibly explaining why RSV incidence in The Netherlands was reported lower than our own. Additionally, the difference in RSV incidence may be due to the use of different methods for the detection of viral pathogen (DFA used in the Netherlands study, and PCR used in our study at Suzhou). Generally speaking, DFA is reported to be less sensitive than that of PCR testing. Surprisingly, the sensitivity of DFA in comparison to rt-RT-PCR was highest (86 %) during the first 3 days after the onset of symptoms, decreasing gradually until reaching 65 % after the first week. The specificity of DFA in comparison to rt-RT-PCR ranged between 99 and 100 % irrespective of the date of collection [12].

In present study, when co-infection existed in conjunction with RSV, IV-A was found the most common co-virus. This is due to the winter/spring seasons seeing the peak of RSV and IV-A activity [13]. To our interest, Mycoplasma pneumoniae was also detected in neonates with LRIs (2.5 %, 45/1803) and 17.8 % (8/45) of those cases were positive for RSV infection. Our recent study suggested that Mycoplasma pneumoniae was also a common cause of bronchiolitis in Suzhou [6]. This indicated that Mycoplasma pneumoniae may be an important pathogen in infants with LRIs.

We further report that RSV incidence increases with age, with RSV infection being more common in late neonates with respect to early ones. We presume that late neonates often have outdoor activities and are susceptible to an increased chance of contracting RSV infection. The increased risk of RSV infection may also be due to the waning of passively derived maternal antibodies. Regardless the cause, the first few days in nursery after birth should be taken into consideration with neonates presenting with LRIs.

In our study, the peak incidence occurred during thewinter and early spring months, as reported in our previous study with a subtropical climate [14, 15] and also expected in a temperateclimate [16, 17]. In our present study, RSV could be detected during the summer months of 2014, which is unusual when compared to other summer months from 2010 to 2013; this phenomenon may be because of low temperatures reported during the summer of 2014, as shown in Fig. 2. However, RSV could circulate throughout summer seasons in Tunisia [10]. This different RSV activity might be caused by the different climate present in Tunisia.

Data generated from previous studies reveal a complex interaction of climate factors especially for temperature and humidity [18–21]. Some of the findings from this studyare similar to others. Monthly mean temperature, sum of sunshine and total rainfall were significantly correlated with the number of RSV-positive neonates with LRIs in this study. Taking the interaction of climate factors into consideration, only mean temperature was associated with RSV activity in neonates. On the contrary, low absolute humidity was independently associated with hospital admission of infants with lower respiratory tract infection due to RSV in northern Spain [21]. In a word, the trend of associations between climate factors and RSV activity varies with geographic locations [22]. In subtropical and temperate regions, RSV incidence is more consistently positively correlated with lower temperatures and higher relative humidity. However, no correlation was found between relative humidity and RSV activity in our study, owing to the stable relative humidity in subtropical Suzhou. Correlations between RSV activity, temperature, and relative humidity arevariable and inconsistent in tropical regions. Further study should be conducted to elucidate the variability between RSV activity and climate factors.

With regard to clinical profiles in neonates with LRIs due to RSV, several studies have focused on the clinical characteristics in neonates with RSV-related LRIs [10, 23–25]. Cho et al. [23] reported thatneonates with RSV-related LRIs had a greater incidence for the requirement of supplemental oxygen after birth, prolonging their hospitalization when compared to neonates infected with other respiratory viruses. As for the chest radiographic pattern in neonates with RSV-related LRIs, RSV infected neonatespresented with more abnormal patterns such as patches, consolidation and atelectasis when compared to non-RSV infected neonates from our study. A recent study [25] reported that neonates with a consolidation pattern on admission had a more severe disease phenotype, with greater risk of invasive mechanical ventilation which is consistent with our study.

To explore the risk predictors of NICU stay in neonateshospitalized with RSVinfection, amultivariable logistic regression model was applied. We found prematurity, coinfection, and underlyingdiseasewere independent risk factors for the severityof RSV infection which is in agreement with other studies [26, 27]. In the Tunisia study [10], prematurebirth and low birthweight might increase therisk of developing severe RSV disease. In contrast to our study, however, low birth weight was not associated with NICU stay. This may be due to the different definitions of low birth weight (2500 VS. 2000 g). Identification of independent risk factors may be contributed to establish a threshold for passiveimmunization and admission to thehospital for high-risk neonates.

## Conclusions

RSV is an important cause of LRIs in hospitalized neonates in Suzhou area with a subtropical climate, particularly during the winter season. Neonates positive for RSV are more prone to severe infection when compared to neonates infected with other common respiratory viruses. The identified predictors ofsevere diseasein neonates with RSV-related LRIs should be taken into consideration when implementing health interventions to newborns.

## Abbreviations

RSV: Respiratory syncytial virus
LRI: Lower respiratory tract infection
CAP: Community-acquried pneumnia
NPA: Nasopharyngeal aspirate
DFA: Direct immunofluorescent assay
PCR: Polymerase chain reaction
IV: Influenza virus
